# Statistical points and pitfalls

**DOI:** 10.1007/s40037-015-0246-0

**Published:** 2016-01-14

**Authors:** Jimmie Leppink, Patricia O’Sullivan, Kal Winston

**Affiliations:** Maastricht University, Maastricht, The Netherlands; University of California, San Francisco, USA; University of Liverpool Online, Amsterdam, The Netherlands

Many readers of this journal are involved in teaching or designing educational programmes or studies. Often, either by yourself or in conjunction with others, you need to design studies asking questions such as what is the best approach for teaching this material for a particular type of learner? What context makes a difference in learner performance? What strategies motivate learners better than others? In planning these studies, there are many design considerations necessary to ensure that the results are meaningful. During the planning of the studies, and subsequently, numerous questions arise concerning the use of statistics. We recognize that medical educational researchers are often not particularly comfortable with statistics and thus could easily slip into making poor decisions about what to do with quantitative data in the study.

Even if your study is designed carefully, choices made inherently lead to a study that has both strengths and limitations. You are now faced with the question of how to present the outcomes of your study and how to formulate implications for educational practice and future research. In this process, you have a diverse toolbox at your disposal from which you need to select tools to match your study, the research questions, and the nature of the data collected. In some types of studies, we tend to make inappropriate data choices [1] either because we are not aware of better alternatives or of how much difference these alternatives can make for the interpretations of findings and the implications thereof for educational practice and future research.

A new section of *Perspectives on Medical Education*, entitled *Statistical points and pitfalls*, directly tackles these analytical issues. The overall purpose of this series is to help readers and researchers alike increase awareness of how to use statistics, why and how we fall into inappropriate choices or interpretations, and what we can do about it. We hope to help readers understand common misconceptions and give clear guidance on how to avoid common pitfalls by offering simple tips to improve the reporting of quantitative research findings. Each entry discusses a commonly encountered inappropriate practice and offers alternatives from a pragmatic perspective with no mathematics involved. This issue’s inaugural edition of *Statistical points and pitfalls* addresses the question of when it is appropriate to do a statistical test. In it, we present the conditions that ought to be met for a statistical test to be used and what to do otherwise [2].

Whether you are an educator trying to select the most appropriate methods to deliver effective learning for your students, a researcher trying to improve your reporting of quantitative findings, or simply want to gain a critical understanding of the literature that you read, this section is for you. If you supervise others’ research but you struggle to give concrete feedback on how to improve, you will also find useful assistance here. Suggestions for future editions of this section can be shared on Twitter, using the hashtag #mededstats. We hope you find *Statistical points and pitfalls* accessible, instructive, and entertaining.
